# PB2 and PA mutations contribute to the pathogenicity of mouse-adapted pdmH1N1-Venus reporter influenza A virus in a mammalian model

**DOI:** 10.3389/fmicb.2024.1532304

**Published:** 2025-01-07

**Authors:** Shixiang Wu, Ruonan Yi, Yingying Tao, Huimin Wu, Li Wu, Jiasheng Song, Xin Zhang, Beibei Yang, Xing Wu, Yulong He, Jianhong Shu, Huapeng Feng

**Affiliations:** ^1^Department of Biopharmacy, College of Life Sciences and Medicine, Zhejiang Sci-Tech University, Hangzhou, China; ^2^Department of Biology, College of Life Sciences, China Jiliang University, Hangzhou, China; ^3^Zhejiang Difference Biotechnology Co., Ltd, Hangzhou, China

**Keywords:** reporter influenza virus, mouse-adapted, pdmH1N1 CA04, Venus, stability, PA and PB2

## Abstract

Influenza A viruses have been a threat to human health for the past 100 years. Understanding the dynamics and pathogenicity of the influenza viruses *in vivo* is of great value in controlling the influenza pandemic. Fluorescent protein-carrying recombinant influenza virus is a substantially useful tool for studying viral characteristics *in vivo* and high-throughput screening *in vitro*. In this study, we generated a recombinant pdmH1N1 CA04 influenza virus carrying a Venus reporter gene in the non-structural (NS) segment using reverse genetics. After passaging the recombinant influenza virus carrying Venus from lung to lung in mice, we found that virulence of the passaged pdmH1N1 CA04-Venus significantly increased and was lethal to the mice. We finally isolated one mouse-adapted pdmH1N1 CA04-Venus with bigger plaques expressing the amount of Venus proteins by using the ninth passage lung homogenate with plague purification. We found three different amino acids (PB2 T340K, PA I21M, and F175L) between WT-CA04-Venus and MA-CA04-Venus using whole-genome sequencing. Interestingly, the polymerase activity of MA-CA04-Venus was significantly lower than that of WT-CA04-Venus in a minigenome assay. Further investigation demonstrates that PA I21M and PA I21M + PB2 T340K significantly enhanced the polymerase activity of WT-CA04-Venus; however, PA F175L + PB2 T340K significantly decreased the polymerase activity of MA-CA04-Venus. Therefore, PA I21M mutation may determine the increased virulence in mice, and PA F175L + PB2 T340K may be involved in the stability of Venus insertion. Above all, we generated a mouse-adapted pdmH1N1 CA04-Venus virus with high virulence and stable green fluorescent Venus protein. It is a useful tool for high-throughput screening of antiviral drugs and for investigating the interaction between the influenza virus and host *in vivo*.

## Introduction

Influenza A viruses (IAVs) belong to the Orthomyxoviridae family and can cause severe respiratory illness, especially in the elderly and children (Fiore et al., 2010). IAVs are categorized according to differences in hemagglutinin and neuraminidase, of which there are 18 and 11 subtypes, respectively (Wille and Barr, 2022). The pdmH1N1/2009 IAVs were first detected in Mexico in 2009 and have rapidly spread worldwide, posing a threat to public health (*MMWR Morb. Mortal Wkly. Rep.,* Centers for Disease Control and Prevention (CDC) 2009a; *MMWR Morb. Mortal Wkly. Rep.,* Centers for Disease Control and Prevention (CDC) 2009b; Sullivan et al., 2010; Yang et al., 2010). In addition, H7N9, H10N8, and other novel influenza viruses (H3N8 and H7N4) continue to pose threats to public health (Wu et al., 2020; Qi et al., 2014). Currently, pdmH1N1 or pdmH1N1-like influenza viruses have become one dominant circulating strain in seasonal influenza. All the seasonal influenza vaccines contain this component to induce the specific immune response. Therefore, understanding the pathogenesis and viral replication mechanisms of pdmH1N1 influenza viruses is an urgent need to prevent and control influenza virus infections.

Although influenza viruses have been extensively studied, the dynamic processes of infection and viral target cells *in vivo* are limitedly understood (Ueki et al., 2018). As new technologies including the *in vivo* imaging system (IVIS) and micro-computed tomography (Micro-CT) developed, real-time detection of the dynamics of the influenza virus infection *in vivo* has been realized. Some researchers have generated multiple replication-competent reporter viruses harboring diverse fluorescent or bioluminescent reporter genes, including eGFP, mCherry, and Gaussia luciferase (Gluc), localized at different segments of the viral genome, mainly in polymerase basic protein 2(PB2), polymerase acidic protein (PA), neuraminidase (NA), and NS segments (Breen et al., 2016; Nogales et al., 2015; Tran et al., 2013; Heaton et al., 2013; Karlsson et al., 2015; Li et al., 2010; Wang et al., 2018). These reporter influenza viruses are powerful tools for identifying antiviral compounds, effective or broadly reactive monoclonal antibodies, or host factors involved in the viral life cycle *in vitro* (Li et al., 2010; Eckert et al., 2014; Munier et al., 2013). Similarly, these reporter expression and replication-competent IAVs are useful to monitor the dynamics of viral infection in tissue culture cells and animal models and test the effect of antiviral drugs and vaccines *in vivo* without invasion (Fukuyama et al., 2015; Pena et al., 2013; Sutton et al., 2014; Kittel et al., 2005). However, there are some limitations among these reporter viruses. The first one is that most replication-competent reporter IAVs were laboratory-adapted strain backbones including A/Puerto Rico/8/34(H1N1, PR8) and A/WSN/1933(H1N1, WSN) viruses (Breen et al., 2016; Manicassamy et al., 2010; Reuther et al., 2015), and the characteristics of these model IAV strains are not always consistent with the current clinical isolates. The second problem is that many reporter IAVs replicate less efficiently than their corresponding parental IAVs, this would affect the use range and make a change compared to the authentic infection dynamics *in vivo* (Pena et al., 2013; Sutton et al., 2014; Manicassamy et al., 2010; Pan et al., 2013). The last aspect is that the foreign genes inserted into the genome of IAVs were partially deleted during the replication and were substantially unstable, especially *in vivo* (Fukuyama et al., 2015; Katsura et al., 2016; Zhao et al., 2015; Furusawa et al., 2019). Therefore, there is an urgent need to generate stable, virulent reporter-expressing, replication-competent IAVs based on the clinical isolate.

In this study, we generated a fluorescent protein expression, replication-competent recombinant A/California/04/2009 (H1N1, CA04) virus harboring the Venus gene in the NS segment, termed as WT-CA04-Venus by reverse genetics. We rearranged the NS segment of CA04 to express non-structural protein 1 (NS1)-Venus and nuclear export protein (NEP) as a polyprotein connecting them by a porcine teschovirus-1 (PTV-1) 2A autoproteolytic cleavage site to allow NEP release from the upstream NS1-Venus protein during translation. Two silent mutations in the splice acceptor site were introduced to avoid the splicing of NS mRNA of CA04. After we passaged the WT-CA04-Venus reporter virus lung to lung nine times, the infected mice presented severe symptoms on day 3 post-inoculation. We purified one mouse-adapted CA04-Venus (MA-CA04-Venus) from the lungs of the ninth passage-infected mice. Then, we characterized this MA-CA04-Venus *in vitro* and *in vivo* and further investigated the molecular determinants of the virulence and stability of this replication-competent reporter virus.

## Materials and methods

### Cells and viruses

Human embryonic kidney epithelial cells (293 T, Cat No. CRL-3216) were obtained from the American Type Culture Collection (ATCC) and maintained in Dulbecco’s Modified Eagle Medium (DMEM; Gibco, New York, United States) containing 10% fetal bovine serum (FBS) with 1% penicillin/streptomycin solution (Gibco, New York, United States). Madin-Darby canine kidney cells (MDCK, Cat. No: CCL-34) obtained from ATCC were maintained in DMEM containing 5% FBS with 1% penicillin/streptomycin (Gibco, New York, United States) in a 37°C containing 5% CO_2_ incubator.

A/California/04/09 (CA04, H1N1) and the CA04 strain carrying the Venus reporter gene (WT-CA04-Venus) were generated by reverse genetics as described previously (Neumann et al., 1999).

### Plasmids

Plasmids for virus rescue were constructed as described previously (Neumann et al., 1999). Eight gene fragments of CA04 were amplified using reverse transcription-PCR, and seven of these fragments were then inserted into the pHH21 vector using BsmBI restriction enzyme digestion. The resulting plasmids were named pHH21-CA04 PB2, pHH21-CA04 PB1, pHH21-CA04 PA, pHH21- CA04 HA, pHH21- CA04 NP, pHH21-CA04 NA, and pHH21-CA04 M. The Venus gene was inserted into the NS segment using overlapping extension PCR and then integrated into the pHH21 vector using homologous recombination, resulting in the plasmid pHH21-CA04 NS-Venus. Next, the ORFs of the polymerase and NP genes (PB2, PB1, PA, and NP) were inserted into the pCAGGS vector, resulting in pCAGGS-CA04 PB2, pCAGGS-CA04 PB1, pCAGGS-CA04 PA, and pCAGGS-CA04 NP. PCR-based targeted mutagenesis was used with primers containing point mutations to generate mutant genes. All plasmids were sequenced to ensure that there were no unwanted mutations.

### Virus rescue

In brief, 293 T cells were seeded into a six-well plate 1 day before transfection. Eight pHH21-vetored recombinant plasmids, four pCAGGS-based recombinant plasmids, and Lipofectamine LTX plus reagent (Thermo Fisher Scientific, United States) were mixed with Opti-MEM (Gibco, New York, United States) and incubated for 20 min at room temperature. Then, the plasmid mixture, along with the transfection reagent, was added to the cells in Opti-MEM (Gibco, New York, United States). At 24 h post-transfection, tosyl sulfonyl phenylalanyl chloromethyl ketone (TPCK) trypsin at a final concentration of 0.5 μg/mL was added. Then, the supernatants and cells were collected at 48 h post-transfection, and the mixture was inoculated into the 10-day-old specific-pathogen-free (SPF) chicken embryonated eggs (Boehringer Ingelheim, Beijing, China) for virus propagation at 35°C. The hemagglutinin test was used to detect the virus, and the full genome was sequenced by Sanger sequencing to avoid unwanted mutations.

WT-Venus-CA04 was continuously passaged in 10-day-old SPF chicken embryo eggs for four generations. Meanwhile, WT-CA04-Venus was passaged in MDCK cells at MOI = 0.01 in MEM (Gibco, New York, United States) supplemented with 0.3% bovine serum albumin (BSA, Sigma, Commonwealth of Pennsylvania, United Kingdom) and 1 μg/mL of TPCK trypsin at 33°C for six times_._ The supernatants and cells were collected when the cytopathic effect (CPE) reached above 80%.

### Passage of the WT-CA04-Venus in the lungs of the mice

To obtain the mouse-adapted CA04-Venus, 6-week-old SPF female BALB/c mice (SLAC, Shanghai, China) were inoculated with WT-CA04-Venus intranasally anesthetized with dry ice; then, the lungs were collected on day 3 post-infection, the lung homogenates were used to infect the naive mice, WT-CA04-Venus was continuously passaged by lung to lung in mice nine times, and the mouse-adapted CA04-Venus strain (MA-CA04-Venus) was selected through plaque purification.

To investigate the pathogenicity of MA-Venus-CA04, five 6-week-old SPF female BALB/c mice per group (SLAC, Shanghai, China) were challenged with 10^5^ PFU/50 μL of either MA-Venus-CA04 or WT-Venus-CA04 via intranasal administration under anesthesia. Body weight and survival were observed continuously for 14 consecutive days.

### Growth kinetics assays

MDCK cells were infected with WT-Venus-CA04 or MA-Venus-CA04 at an MOI of 0.01 in triplicate and were incubated at 33°C or 37°C, respectively, with MEM containing 0.3% BSA (Sigma, Commonwealth of Pennsylvania, United Kingdom) with 1 μg/mL of TPCK trypsin. The supernatants were collected at 12 h, 24 h, 36 h, 48 h, 60 h, and 72 h post-infection. Subsequently, the viral titers in the supernatant at each time point were determined using a plaque assay.

### Minigenome assay

pCAGGS-CA04 PB2, pCAGGS-CA04 PB1, pCAGGS-CA04 PA, and pCAGGS- CA04 NP from WT-CA04-Venus or MA-CA04-Venus (0.25 μg each) and plasmids expressing firefly luciferase protein (pPolI-NP(0)Luc(0), 0.25 μg) and expressing *Renilla* luciferase gene (pRL-null, 0.25 μg) used as internal control were co-transfected into 293 T cells with Lipofectamine LTX and Plus reagent (Thermo Fisher Scientific, United States). At 48 h post-transfection, the supernatants were removed, and the cell lysates were prepared. The activity of firefly luciferase and *Renilla* luciferase was detected using the Dual-Glo luciferase assay system and measured using a GloMax 96 microplate Luminometer (Promega, California, United States; Feng et al., 2022).

### Statistical analysis

Student’s *t-test* was used to perform the statistical analysis using GraphPad Prism 6.0 (GraphPad Software Inc., San Diego, CA, United States). A *p-value of* < 0.05 indicates a statistically significant difference.

## Results

### Rescue of the pdmH1N1 virus carrying the Venus reporter gene in the NS segment

**Figure 1:**
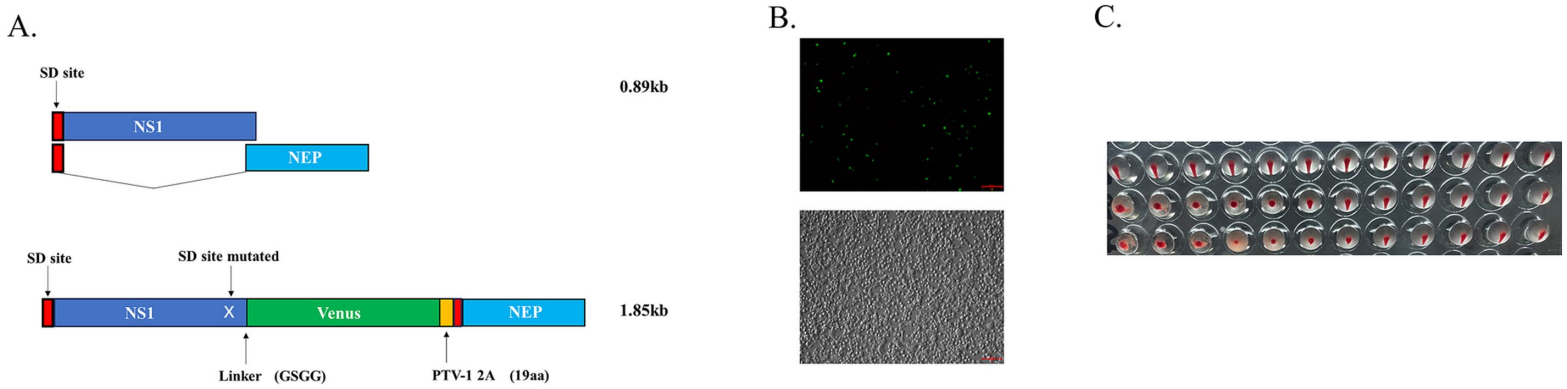
Generation of recombinant CA04 virus carrying the Venus reporter gene. **(A)** Schematic map of CA04 NS carrying the Venus reporter gene. The splicer acceptor site (SA) in NS is mutated to prevent mRNA splicing. NS1 (dark blue); NEP (light blue), splice donor site (SD, red), NS1 is fused to Venus (green) via a linker (GSGG) followed by the PTV-1 2A autoproteolytic cleavage site (yellow); **(B)** Twelve plasmid transfected 293 T cells. Fluorescent protein expression was detected at 48 h post-transfection. **(C)** Cells and cell culture supernatants were inoculated with chicken embryos and hemagglutinated after 72 hpi.

The Venus fluorescent reporter gene, a GFP variant with eight mutations, was effective in improving chromophore formation and fluorescence intensity (Fukuyama et al., 2015). In this study, we inserted the Venus reporter gene into the C-terminus of the NS1 of the pdmH1N1 CA04 influenza virus and fused the junction through the GSGG linker; meanwhile, two mutations were introduced in a splicer acceptor site (SA) to prevent being spliced by mRNA processing (Figure 1A). Forty-eight hours post-transfection, the cells were first detected using fluorescent microscopy, and the supernatants and the cells were collected (Figure 1B). Subsequently, the mixture was inoculated into the 10-day-old embryonated eggs. At 72 h post-inoculation, the harvested allantoic fluid exhibited hemagglutinating activity (Figure 1C). Sanger sequencing was performed on the full genome of WT-CA04-Venus and confirmed that there were no unwanted mutations.

### Characterization of WT-CA04-Venus virus *in vitro*

**Figure 2:**
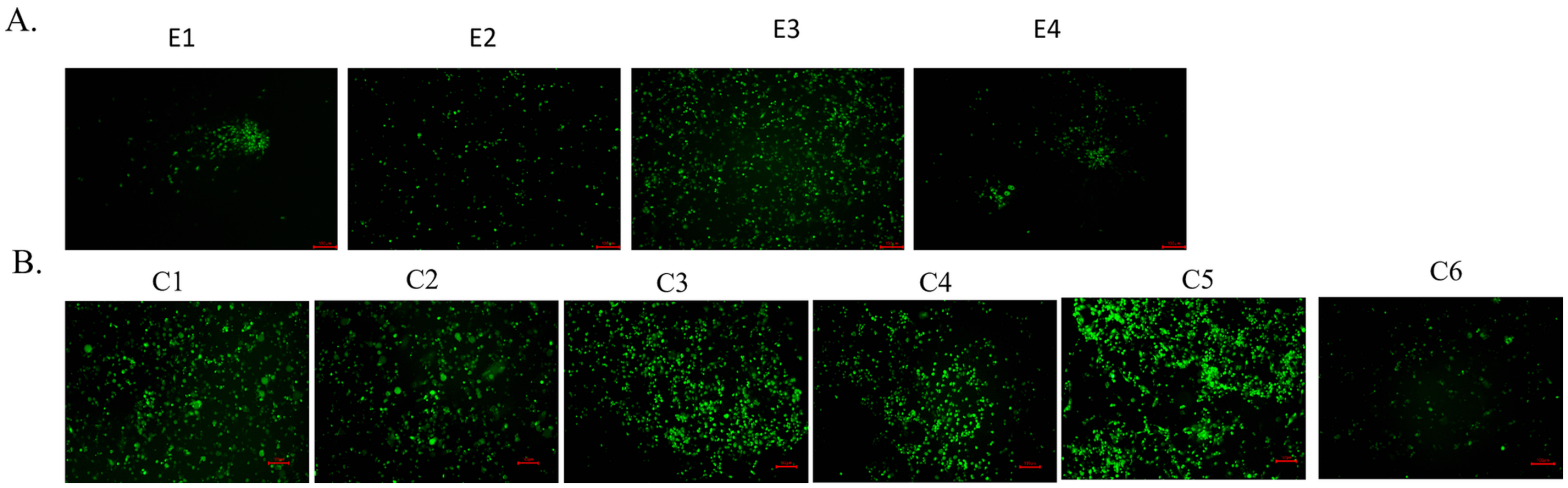
Passaging of WT-CA04-Venus in chicken embryos and mammalian cells. **(A)** Passaging of WT-CA04-Venus in chicken embryos. WT-CA04-Venus virus was serially diluted 10 times, the SPF eggs were inoculated in a 200 μL volume from 10^−3^ to 10^−5^ dilution into the allantoic cavity for three eggs per dilution and incubated at 35°C for 72 h; then, we repeated the above process. E1–E4 generation viruses were inoculated into MDCK cells with a final concentration of 1 μg/mL of TPCK trypsin, and the fluorescence expression was observed after 24 h post-infection. **(B)** Passaging of WT-CA04-Venus in MDCK cells. Fluorescence expression profile of WT-CA04-Venus in six consecutive passages in MDCK cells. In brief, MDCK cells were infected with P0 of the WT-CA04-Venus at an MOI of 0.01, and the infected cells were incubated at 33°C. With a final concentration of 1 μg/mL of TPCK trypsin, the fluorescence expression was observed after 48 h post-infection. We collected the supernatant when the CPE reached over 80% and then infected new MDCK cells to passage the recombinant virus up to six times.

Many influenza viruses with different fluorescent reporter genes and locations have been generated (Eckert et al., 2014; Fukuyama et al., 2015; Bu et al., 2021; Nogales et al., 2019). However, these influenza viruses carrying fluorescent reporter genes are highly susceptible to loss of foreign genes (Manicassamy et al., 2010). At first, WT-CA04-Venus was continuously passaged in chicken embryonated eggs up to four times, and these four generations of viruses were infected with the same dose in MDCK cells, and fluorescent protein expression was observed in all four generations at 24 h post-infection. However, less fluorescent expression was observed in the fourth generation (Figure 2A). WT-CA04-Venus was stably present in chicken embryos for the first several generations, but the stability of its fluorescent proteins decreased with the increase in the number of passages. MDCK cells were infected for six consecutive passages, and the fluorescence expression was observed; it was found that the fluorescence intensity in the sixth generation was lower than that in the first five generations. So possibly, there was a partial loss of fluorescent protein in the sixth generation of WT-CA04-Venus (Figure 2B). Therefore, WT-CA04-Venus was able to maintain the stability of the fluorescent gene inserted into the genome over the limited passages *in vitro*.

### Mouse-adapted CA04-Venus was generated by the lung-to-lung passage in mice and plaque purification

**Figure 3:**
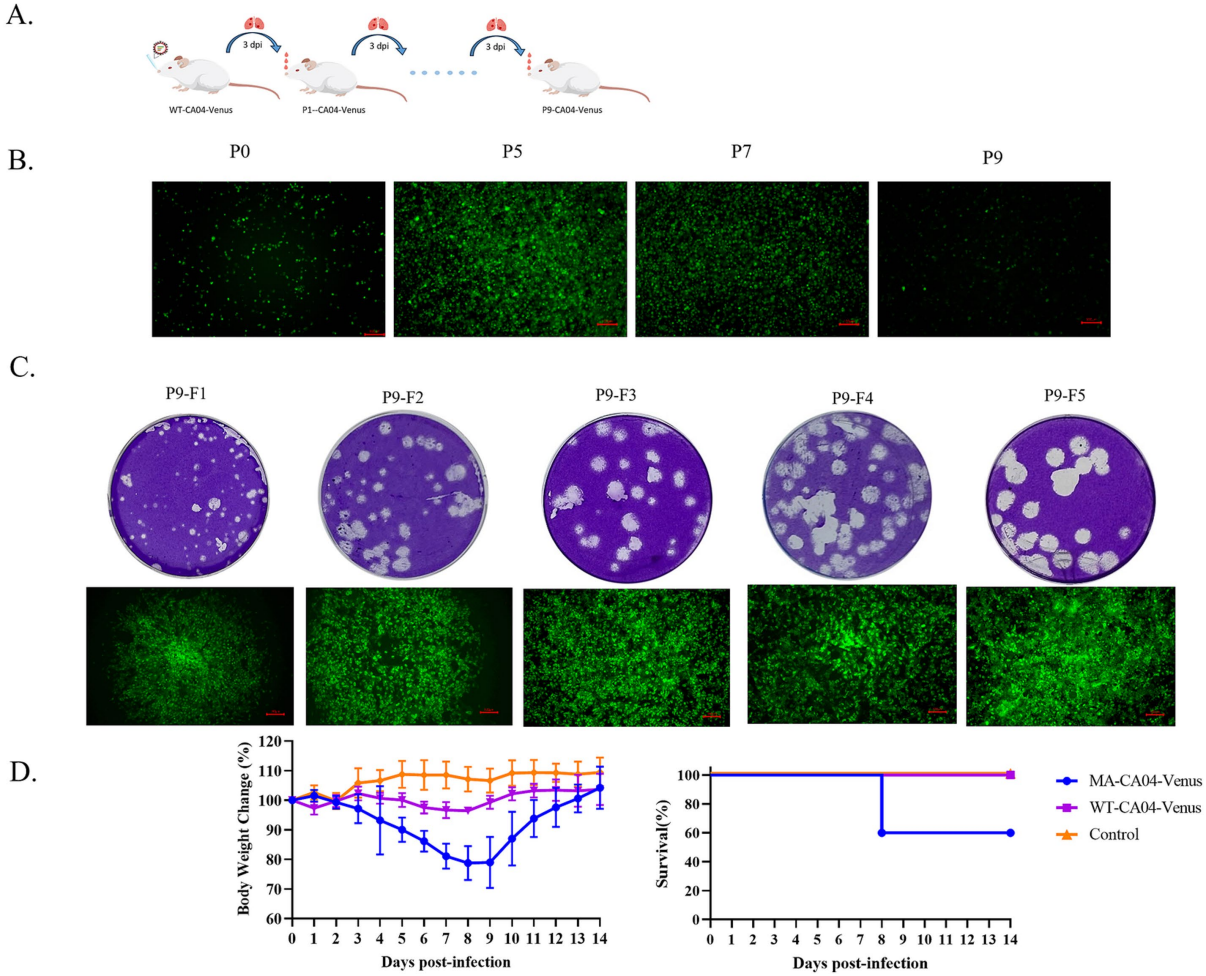
Generation and characterization of mouse-adapted CA04-Venus recombinant virus. **(A)** Schematic diagram of passaging WT-CA04-Venus in the lungs of the mice. BALB/c mice were anesthetized with dry ice; then, 50 μL of the virus was dropped intranasally, and lungs were harvested on day 3 post-infection. The lung homogenate was then used to infect the naive mice until nine times. **(B)** The MDCK cells were infected with lung homogenates of different passages, and the Venus expression was detected at 24 h post-infection. **(C)** Mouse-adapted CA04-Venus was purified from the lung homogenates of the ninth passage by five-round purification. **(D)** Body weight changes and survival of the mice infected with WT-CA04-Venus or MA-CA04-Venus. The mice were infected with 10^5^ PFU by intranasal administration, and the body weight and survival were monitored each day for 14 days.

PdmH1N1 CA04 influenza virus is low pathogenic to the mice (Itoh et al., 2009). To generate a mouse-adapted CA04-Venus strain, we continuously passaged the WT-CA04-Venus through lung-to-lung passages as indicated in Figure 3A. The body weight began to decrease from the fifth passage, and the WT-CA04-Venus was continuously passaged until the ninth passage. The infected mice with the ninth passage lung homogenates presented significant body weight loss on day 3 post-infection.

As stated above, WT-CA04-Venus is highly susceptible to fluorescent protein loss during passaging in mice. Therefore, MDCK cells were inoculated every other generation from the fifth passage of CA04-Venus (Figure 3B). The fluorescent protein was expressed with the ninth passaged lung homogenates while significant weight loss was also presented in mice, so we purified the bigger plaque with strong intensity of the Venus fluorescent reporter protein for five rounds when the fluorescence positivity reached 100% and no fluorescent protein was lost by using ninth passaged lung homogenates. Finally, we got one mouse-adapted CA04-Venus strain, termed MA-CA04-Venus (Figure 3C). To confirm the virulence of the MA-CA04-Venus in mice, we infected the mice with MA-CA04-Venus or WT-CA04-Venus virus by intranasal inoculation and the body weight and survival was detected every day for 14 days. We found that the body weight of the mice infected with MA-CA04-Venus exhibited significant body weight loss and two of the five infected mice were dead while the mice infected with WT-CA04-Venus only presented a slight body weight loss (Figure 3D). These results demonstrated that MA-CA04-Venus was significantly more virulent than that of the WT-CA04-Venus.

### The replication ability of the MA-CA04-Venus was significantly enhanced in mammalian cells

**Figure 4:**
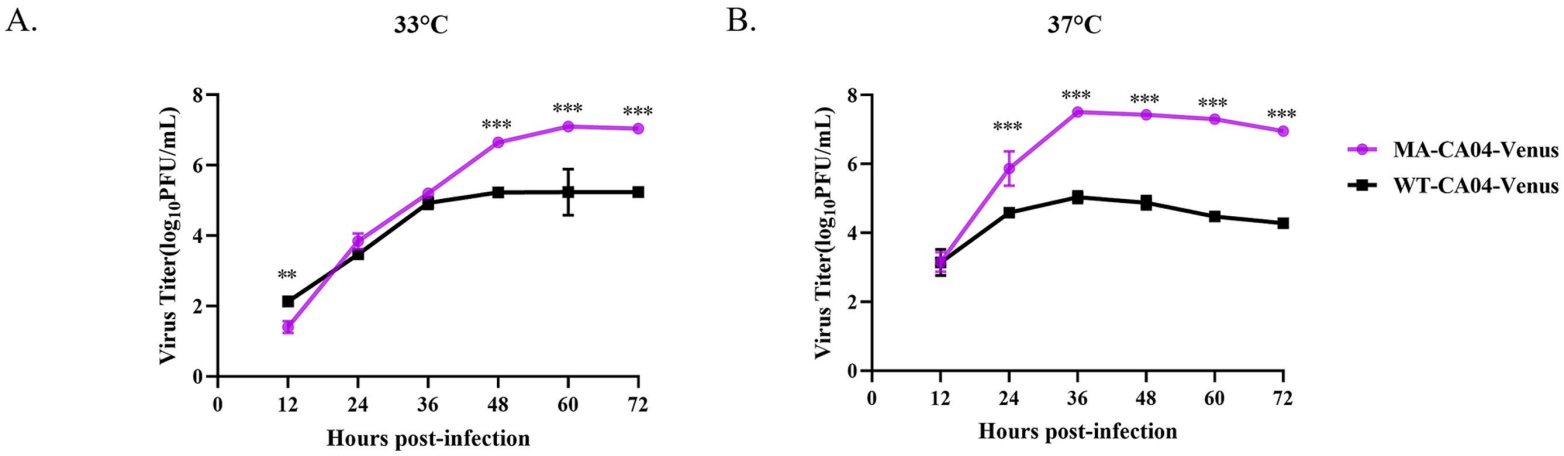
Growth kinetics of WT-CA04-Venus and MA-CA04-Venus in MDCK cells. **(A)** Growth kinetics of WT-CA04-Venus and MA-CA04-Venus in MDCK cells at 33°C, **(B)** growth kinetics of WT-CA04-Venus and MA-CA04-Venus in MDCK cells at 37°C. MDCK cells were infected with the indicated recombinant influenza viruses at an MOI of 0.01, and cell supernatants were collected every 12 h until 72 h post-infection. The viral titers were determined by plaque assay. The data shown are in triplicate, and the values are means ± standard deviation. **p < 0.01 Comparison with WT-CA04-Venus viruses. Ns, not significantly different. ***p < 0.001 Comparison with WT-CA04-Venus viruses. Ns, not significantly different.

To investigate the replication ability of MA-CA04-Venus in mammalian cells, we infected the MDCK cells with WT-CA04-Venus or MA-CA04-Venus and incubated the infected cells at 33°C or 37°C, respectively. The supernatants were collected at the indicated time points, and the viral titers were determined using a plaque assay. We found that the viral titers of MA-CA04-Venus were significantly higher than those of WT-CA04-Venus at both 33°C and 37°C in general. The viral titers of these two viruses were similar at 24 h post-infection (hpi) at 33°C, but the viral titers of MA-CA04-Venus were significantly higher than those of WT-CA04-Venus at 48, 60, and 72 hpi at 33°C (Figure 4A). The viral titers of MA-CA04-Venus were higher than those of WT-CA04-Venus at 24, 36, 48, 60, and 72 hpi at 37°C (Figure 4B). These results were consistent with high virulence of MA-CA04-Venus in mice.

### Amino acid mutations in PB2 and PA polymerase proteins contribute to the adaptation of MA-CA04-Venus in mice

**Figure 5:**
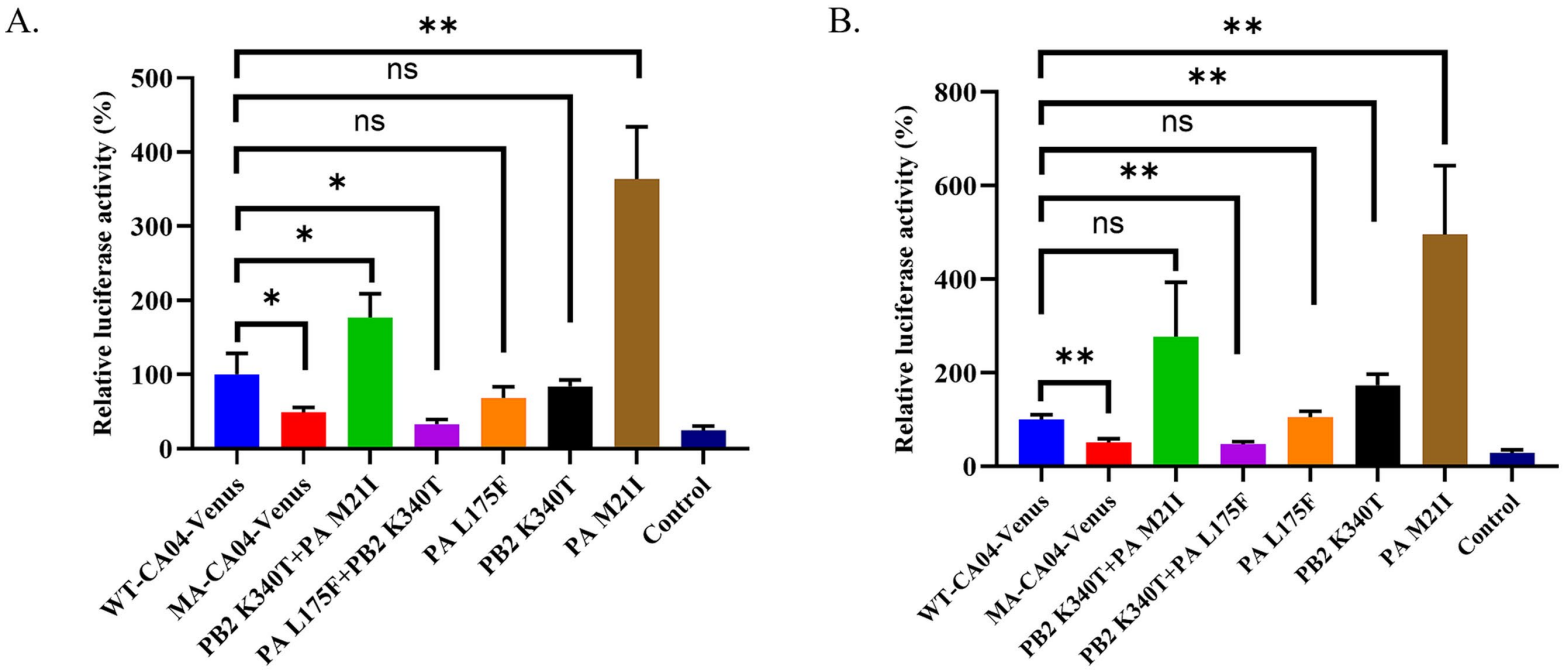
Polymerase activity of different RNP combinations derived from the WT-CA04-Venus or MA-CA04-Venus viruses. 293 T cells were transfected in triplicate with a luciferase reporter plasmid and an internal control plasmid, together with plasmids expressing PB2, PB1, PA, and NP from either WT-CA04-Venus or MA-CA04-Venus virus. **(A)** The transfected cells were incubated at 33°C for 24 h. **(B)** The transfected cells were incubated at 37°C for 24 h, and cell lysates were analyzed for firefly and *Renilla* luciferase activities. The values shown are the mean ± standard deviation of data from three independent experiments and normalized to the activity of WT-CA04-Venus (100%). *p < 0.05. Comparison with WT-CA04-Venus virus. **p < 0.01. Comparison with WT-CA04-Venus viruses. Ns, not significantly different.

To underlie the mechanism of MA-CA04-Venus possessing the high replication ability in mammalian cells and high virulence in mice, we compared the full-genome sequences between WT-CA04-Venus and MA-CA04-Venus and found that there are three amino acid substitutions (PB2 K340T, PA M21I, and PA L175F; Table 1). The mutations are mainly located at the PB2 and PA subunits of the polymerase of the influenza virus. Therefore, we compared the polymerase activity of WT-CA04-Venus and MA-CA04-Venus viruses and detected the effect on polymerase activity of one amino acid mutation (PB2 K340T, PA M21I, and PA L175F) and double amino acid substitutions (PB2 K340T + PA M21I and PB2 K340T + PA L175F) using a minigenome assay. Surprisingly, the polymerase activity of MA-CA04-Venus was significantly lower than that of WT-CA04-Venus at both 33°C and 37°C in 293 T cells (Figures 5A,B). PA M21I significantly enhanced the polymerase activity of WT-CA04-Venus up to 3.6-fold at 33°C and 4.9-fold at 37°C. PA L175F did not affect the polymerase activity of WT-CA04-Venus. PB2 K340T did not change the polymerase activity at 33°C, while this mutation significantly enhanced the polymerase activity WT-CA04-Venus at 37°C. PB2 K340T + PA M21I double mutations significantly increased the polymerase activity at 33°C, while the difference was not significant at 37°C. However, PB2 K340T + PA L175F double substations significantly decreased the polymerase activity of WT-CA04-Venus at both 33°C and 37°C, which was similar to that of MA-CA04-Venus (Figures 5A,B). Therefore, it suggests that the PA M21I resulted in a significant increase in the polymerase activity of the influenza virus, while PB2 K340T + PA L175F double amino acid substitutions caused a decrease in the viral polymerase activity. These results indicate that PA M21I was involved in the high virulence of the MA-CA04-Venus while PB2 K340T + PA L175F was associated with maintaining the stability of Venus in the viral genome of the influenza virus.

**Table 1:** Amino acid differences between WT-CA04-Venus and MA-CA04-Venus in PB2 and PA.

Viral proteins	Amino acid position	Amino acids	
		WT-CA04-Venus	MA-CA04-Venus
PB2	340	K	T
PA	21	M	I
	175	L	F

## Discussion

Influenza viruses harboring the reporter genes including the fluorescent or luciferase proteins are substantially useful for the high-throughput screening for antiviral drugs, invasion visualization of dynamics of influenza virus replication *in vivo*, and virus–host interaction. However, the insertion of foreign genes into the genome of influenza viruses often results in decreased pathogenicity and less stability of reporter genes, especially for seasonal influenza viruses (Heaton et al., 2013; Manicassamy et al., 2010). In this study, we first generated the WT-CA04-Venus recombinant influenza virus carrying the Venus fluorescent reporter gene in the NS segment using reverse genetics, and then, one mouse-adapted CA04-Venus was obtained by the lung-to-lung passage in mice and plaque purification. MA-CA04-Venus was able to maintain its stability in limited passages by successive passages in SPF embryonated eggs and mammalian cells *in vitro* (Figures 2A,B). MA-CA04-Venus grew better compared to that of WT-CA04-Venus (Figure 4). Further investigation demonstrates that there are three amino acid substitutions in the genome of MA-CA04-Venus compared to that of WT-CA04-Venus including PB2 K340T, PA M21I, and PA L175F (Table 1). We found that the PA M21I significantly enhanced the polymerase activity of the influenza virus, while PB2 K340T + PA L175F double mutations decreased the viral polymerase activity. Therefore, in this study, we generated a mouse-adapted CA04-Venus stably harboring the Venus fluorescent gene with good replication ability *in vitro* and relatively high virulence in mice and further explored the molecular mechanism of maintaining high virulence and stabilizing the reporter gene.

The rescued recombinant influenza viruses carrying the fluorescent proteins or luciferase often presented a lower replication ability *in vitro* and attenuated pathogenicity *in vivo* (Pena et al., 2013; Sutton et al., 2014; Manicassamy et al., 2010; Pan et al., 2013); Pan et al. generated the recombinant PR8 influenza virus harboring the Gluc in the NA segment, PR8-Gluc. They found that PR8-Gluc replicated lower than that of WT-PR8 virus in both SPF embryonated eggs and MDCK cells, and compared to its counterpart, the virulence of PR8-Gluc in mice was attenuated up to 1,000 times (Pan et al., 2013). Fukuyama et al. generated multiple Color-flu influenza viruses carrying different fluorescent proteins (e.g., eGFP, eCFP, mCherry, and Venus) and found the recombinant fluorescent influenza viruses (e.g., WT-PR8-Venus and WT-H5N1-Venus) were significantly attenuated in mice. These attenuated viruses are suitable for antiviral screening *in vitro*, but they could not reflect the real immune responses when infecting mice or other animals. Subsequently, these fluorescent influenza viruses obtained higher pathogenicity after serial passage in the lungs of the mice. However, they did not generate the fluorescent current seasonal influenza viruses in their study (Pena et al., 2013). In the current study, we generated the recombinant pandemic seasonal H1N1 influenza viruses expressing the Venus protein and obtained a higher pathogenic MA-CA04-Venus reporter virus through serial lung passage in mice. These demonstrate that the attenuation resulting from the insertion of foreign genes may not be associated with the types of fluorescent proteins and the backbones of influenza viruses. Serial passage in the lungs of the mice is an effective and broad applicable strategy to solve this problem.

The selection of fluorescent proteins or luciferases is dependent on the applications of the replication-component influenza viruses. Recombinant IAV-expressing fluorescent proteins are suitable for the study of cellular localization and virus–host cell interaction *ex vivo* due to their penetrability (Chiem et al., 2019). Recombinant IAV-expressing luciferases are better for the quantification and detection of the infection progress *in vivo* by the IVIS system; however, bioluminescence-expression reporter virus requires systemically injection of the substrates before every observation; this may interfere with the immune responses, and we cannot trace target cells infected with influenza viruses using the luciferase-expressing recombinant influenza viruses (Breen et al., 2016). As we know, EGFP is the most versatile fluorescent protein used in biology and imaging. Venus is derived from the YFP with five amino acid substitutions and eight amino acids difference compared to EGFP; however, Venus is brighter up to 30-fold than the YFP with the same spectrum. On the other hand, Venus has a narrower spectrum than EGFP and does not easily overlap with other fluorescent proteins when multicolor experiments (Nagai et al., 2002; Okita et al., 2004). PR8 influenza virus expressing the Venus combined with two-photo microscopy has been used to trace the movability of the neutrophil cells labeled Ly6G with Cy3 in the lungs of the infected mice (Okita et al., 2004). Therefore, we select Venus as a fluorescent protein to generate the replication-competent seasonal reporter influenza virus in our study.

Underlying the mechanisms of the virulence and stability of the recombinant influenza viruses expressing the foreign genes would be useful for broadening our knowledge of the viral life cycle and the development of novel influenza virus-vectored vaccines. HA T380A and PB2 E712D enhanced the viral replication and pathogenicity in mice of the PR8 Venus-expressing reporter influenza virus, and PB2 E712D plays a crucial role in the enhancement (Eckert et al., 2014). To further analyze the mechanism, Furusawa et al. found that PB2 E712D contributes to the stability of Venus insertion by improving the efficiency of the modified NS segment (Sakabe et al., 2011). Zhao et al. found that PB2 V25A combined with PA R443K significantly enhanced the pathogenicity of the WT-Venus-H5N1 virus carrying the modified NS segment of PR8 in mice (Zhao et al., 2015). From the above studies, we know that mutations in the polymerase complex of the influenza virus must occur to enhance the pathogenicity in mice and stabilize the insertion of foreign fluorescent genes into its genome. In this study, PB2 K340T, PA M21I, and PA L175F of WT-CA04-Venus were presented during the serial passage in mice. PA M21I mutation was reported to enhance the virulence of the CA04 influenza virus in a previous study (Sakabe et al., 2011). We found that PA M21I mutation substantially enhanced the polymerase activity of WT-CA04-Venus (Figure 5). Therefore, PA M21I mutation is a virulent determinant of MA-CA04-Venus. In the other aspect, the polymerase complex plays a key role in the replication and genome stability of the influenza virus. In this study, we found the polymerase activity of MA-CA04-Venus was significantly lower compared with that of WT-CA04-Venus (Figure 5). Minigenome assay demonstrated that PB2 K340T and PA L175F double mutations contribute to the lower polymerase activity of MA-CA04-Venus. In the previous study, the polymerase activity of MA-Venus-PR8 or MA-H5N1-Venus was significantly reduced compared to their respective WT counterparts. The PB2 E712D substitution was found to reduce polymerase activity and influence the stability of the foreign gene inserted into the viral genome of MA-Venus-PR8. In contrast, PB2 V25A and PA R443K contributed to the reduced polymerase activity and enhanced stability of Venus in the MA-H5N1-Venus after serial passage in mice (Eckert et al., 2014; Sakabe et al., 2011). Therefore, PA M21I substitution is responsible for the high virulence, and PB2 K340T and PA L175F double mutations contribute to the stability of the Venus gene in the NS segment of MA-CA04-Venus.

In conclusion, we generated recombinant pdmH1N1 CA04 reporter influenza virus expressing the Venus fluorescent protein, WT-CA04-Venus. This recombinant influenza virus could maintain the stability of the Venus gene inserted into the genome in the limited passages *in vitro*. Mouse-adapted CA04-Venus was further generated through serial passage in the lung of the mice and five rounds of plaque purification, and MA-CA04-Venus presented more bright fluorescence, higher growth ability, and higher virulence compared to WT-CA04-Venus. Further investigation demonstrates that PA M21I is a potential virulent determinant, and PB2 K340T and PA L175F mutations are responsible for the enhanced stability of the Venus gene inserted into the genome of MA-CA04-Venus. These results broadened our knowledge of the replication of the influenza virus and provided a powerful tool to explore the virus–host interaction, evaluate the efficacy of the antiviral drugs and broadly reactive monoclonal antibody, and detect the viral infection dynamics caused by the 2009 H1N1 influenza virus combined with novel imaging technology.

## Data Availability

The original contributions presented in the study are included in the article/supplementary material, further inquiries can be directed to the corresponding author.
